# Clinical characteristics that portend a positive Xpert Ultra test result in patients with pleural tuberculosis

**DOI:** 10.7196/AJTCCM.2019.v25i2.011

**Published:** 2019-07-31

**Authors:** E Makambwa, H R Maboreke, M Fadul, R Meldau, M Dhansay, A Esmail, K Dheda

**Affiliations:** 1 Centre for Lung Infection and Immunity, Division of Pulmonology, Department of Medicine and UCT Lung Institute, University of Cape Town, South Africa; 2 London School of Hygiene and Tropical Medicine, London, United Kingdom

**Keywords:** pleural TB

## Abstract

**Background:**

The performance of Xpert-MTB/RIF, including the newer Xpert Ultra test, for the diagnosis of pleural TB is poor (~28 - 38%). There are no data on patient characteristics that portend a positive Xpert-Ultra test in pleural fluid. These characteristics could be useful for selecting patients for Xpert-Ultra testing, thus maximising benefits of a positive test, while minimising cost.

**Objectives:**

To determine the clinical, radiological, microbiological and biochemical characteristics associated with Xpert-Ultra positivity in patients with suspected pleural tuberculosis.

**Methods:**

We performed a subgroup analysis of a prospective observational cohort (N=165 patients with suspected pleural TB) evaluating same-day diagnostic tools for pleural tuberculosis. Forty-nine patients with confirmed pleural tuberculosis (culture and/or histology) were included in the final analysis.

**Results:**

Of the 49 participants, 17 (35%) were female and 9 (18.4%) were HIV-infected. In the multivariate analysis including demographic, radiological and pleural fluid test characteristics, there were no independent predictors of Xpert-Ultra positivity. However, when pleural fluid test results were excluded, and when only rapidly ascertainable pre-test factors (demographic and radiologic variables) were considered, the multivariable analysis showed that only a chest X-ray (CXR) suggestive of active TB (cavities, consolidation and hilar lymphadenopathy) was associated with Xpert-Ultra positivity (p=0.021). Notably, only 22% (n=11/49) of the participants had a CXR suggestive of active TB and of these, 73% (n=8/11) had a positive Xpert-Ultra result.

**Conclusion:**

CXR features suggestive of active TB are significantly associated with a positive Xpert-Ultra test result on pleural fluid. These data inform clinical practice in resource-poor settings.

## Background

Tuberculosis (TB) remains a public health crisis in sub-Saharan Africa where it is a leading cause of death.^[1,2]^ Extrapulmonary TB (EPTB) comprises 15% of active TB cases, although in HIV endemic countries this figure is closer to 42%.^[3]^ The diagnosis of extra-pulmonary TB remains a major challenge due to the paucibacillary nature of the disease and the need for invasive procedures to obtain samples.^[4,5]^

The frontline diagnostic test for the diagnosis of pulmonary TB is the Xpert Ultra. This test performs well on sputum with an estimated sensitivity of ~70 - 80% in smear negative disease.^[6]^ However, its performance in pleural TB is underwhelming, with an estimated sensitivity of ~30%.^[7,8,9]^ Furthermore, Meldau *et al*. (unpublished data) have recently shown, in 165 patients with suspected pleural TB, that Xpert Ultra is only slightly better than the Xpert MTB/RIF (G4 cartridge) with a sensitivity of ~37% for the diagnosis of pleural TB. Thus, Xpert is a poor test choice for the diagnosis of pleural TB, especially when cost is taken into consideration.

Despite the low sensitivity of Xpert in pleural fluid, a positive test result would be useful for the clinician, especially in a high MDR-TB prevalence setting, as it provides information on the susceptibility of *Mycobacterium tuberculosis* to rifampicin. However, there are no data on patient characteristics that portend a positive Xpert test in pleural fluid. These characteristics could be useful for selecting patients for Xpert Ultra testing, thus maximising benefits of a positive test while minimising costs. It is particularly important for any predictive characteristics to be rapidly and easily identifiable prior to pleural fluid testing so that the appropriate testing strategy can be optimally selected. We therefore set out to determine the clinical, radiological, microbiological and biochemical factors that predict patients that will probably test positive with Xpert Ultra. We further analysed demographic and radiological variables separately for their predictive value, given that this is the only information that can be rapidly ascertained prior to any pleural fluid testing.

## Methods

### Study design and patient recruitment

The parent study prospectively recruited 165 patients with suspected pleural TB (any TB symptoms and features consistent with a pleural effusion on CXR (CXR)) from Groote Schuur Hospital in Cape Town, South Africa, over a 9-year period (2009 - 2018) to evaluate tools for the rapid diagnosis of pleural TB (Meldau and Dheda, unpublished data). Due to limitations of a single pleural fluid culture for confirming a diagnosis of pleural TB, a composite reference standard was used for patient categorisation. Definite TB was defined as at least one positive *M. tuberculosis* culture (pleural fluid and/or biopsy) and/or caseating granulomatous inflammation suggestive of TB on histological examination of pleural biopsy tissue, and with improvement on anti-TB treatment (all patients with definite TB received anti-TB treatment). There were 49 patients with definite TB and all of them were included in this subgroup analysis.

### Demographic characteristics

Clinical information (age, sex, HIV status and CD4 count) was extracted from the research database and patient source documents. Previous TB was reported by the patient and in most cases corroborated by medical records.

### Pleural fluid tests

Microbiological (Z-N stain, pleural fluid culture and time to positive culture (TTP)), biochemical (pleural fluid protein and adenosine deaminase (ADA)) and Xpert Ultra tests were performed.

### Radiological characteristics

For the purpose of this study, effusions occupying less than onethird, one-third to two-thirds and greater than two-thirds of hemithorax were characterised as small, moderate and large, respectively, on PA films.^[10]^ Presence of cavities, consolidation and hilar lymphadenopathy on CXR were defined as features suggestive of active TB. CXR records between 2012 and 2018 were retrieved from the iSite Enterprise electronic system while the plain CXRs done between 2009 and 2012 were retrieved from medical records.

### Statistical analysis

As the objective was to ascertain the predictive value of Xpert Ultra in those patients with pleural TB, the analysis was restricted to 49 definite TB cases. Medians and interquartile ranges (IQRs) were calculated for continuous variables and frequency (percent) for categorical variables. Comparisons of continuous variables were performed using Wilcoxon rank sum tests while χ² tests were used for categorical variables. Logistic regression analysis included variables that were significantly associated with the Xpert Ultra result. A significance value of p <0.05 was used. Statistical analyses were done in Stata version 13 (StataCorp., USA).

## Results

### Univariate and multivariate analysis using all variables

Overall, 35% of our study population was composed of females, while 18.4% of the participants had HIV co-infection. Individual patients’ characteristics and their association with the Xpert ultra test have been summarised in Table 1. Pleural fluid tests such as pleural fluid protein (p=0.01) and ADA (p=0.001) showed individual predictive powers for a positive Xpert Ultra result. Furthermore, pleural effusion size (0.06) and time to positive culture (p=0.09) showed a trend towards significance.

In a model including all variables with p<0.1, individual characteristics lost significance on multivariate analysis as shown in Table 2. ADA was the only variable that showed a trend towards significance (p=0.08).

### Univariate and multivariate analysis of immediately ascertainable pre-test variables (demographic and radiological variables)

In the univariate analysis, CXR suggestive of active TB was the only rapidly ascertainable variable that was predictive of a positive Xpert Ultra result (p=0.01). More importantly, in the multivariate analysis, CXR suggestive of active TB (p=0.021) was associated with a positive Xpert Ultra result (Table 3).

### Xpert Ultra results in association with CXR findings

More than a quarter of the patients (27.3%; n=11/49) had a CXR suggestive of active TB. Of these, 72.7% (n=8/11) had a positive Xpert Ultra result (Table 4). Similarly, 66.7% (n=6/9) patients with a large pleural effusion size had a positive Xpert Ultra result (Table 1).

## Discussion

We evaluated clinical characteristics that predict a positive Xpert Ultra test result in pleural fluid. The key finding of this study was that CXR was an independent predictor of Xpert Ultra positivity when taking into account immediately ascertainable pre-test variables. This has obvious implications for selection of tests and costs.

In the multivariate analysis using immediately ascertainable factors, CXR was the only independent predictor of Xpert positivity. On reflection, this finding is perhaps not surprising, given that patients with radiological features of active TB are likely to have parenchymal lung involvement. Indeed, 72.7% of patients in our study with features suggestive of active TB had a positive Xpert Ultra result. It is important to note that only 27.3% of patients had such CXR characteristics, thus limiting the utility of this approach. However, looking at this from a different angle would suggest that in a quarter of patients, this strategy may still be useful by allowing cost-effective usage of Xpert Ultra in TB-endemic countries. Indeed, in many countries such as Malawi and Mozambique, Xpert Ultra is not readily available and is used selectively. This approach will be very useful in such settings. To the best of our knowledge, this is the first time that a readily available investigation uch as a CXR, often available prior to pleural fluid aspiration and testing, has been shown to predict Xpert ultra positivity on pleural fluid.

In the univariate model using all the characteristics (including immediately ascertainable factors and pleural fluid test results), pleural effusion size (p=0.06) and time to positive culture (p=0.09) showed a trend towards significance. Patients with a large pleural effusion were more likely to test positive for Xpert Ultra, suggesting a larger burden of disease compared with those patients with small or moderate effusion size. Similarly, time to positive culture (TTP), which is a surrogate of mycobacterial load, was shorter in those with positive Xpert Ultra compared to those who had a negative test (18.8 days v. 23 days, respectively). Therefore, patients with a higher disease burden (borne out of a lower TTP and large pleural effusion size) were more likely to test positive for Xpert Ultra.

When all characteristics with p<0.1 were included in a multivariate model, individual characteristics lost their significance. This was surprising given that pleural fluid ADA and pleural fluid protein are well recognised for their role in facilitating a TB diagnosis.^[11,12]^ However, in our model, ADA only showed a trend towards significance (p=0.08). This might be an effect of our small sample size. In any event, waiting for an ADA result (not rapidly ascertainable prior to any pleural fluid testing) will result in unnecessary delay and will require aspiration and diagnostic testing in its own right, which is also resource-consuming.

HIV status was not significantly associated with a positive Xpert Ultra result. Given the impaired cellular immunity in HIV-infected participants, one would expect a higher disease burden and hence a higher likelihood of a positive test. This was not the case in our study. In a study on extrapulmonary TB in a setting of HIV hyperendemicity in Durban, South Africa, Gounden *et al*. ^[3]^ showed that HIV co-infection was a common risk factor for extrapulmonary TB, suggesting impaired cellular immunity as an explanation for the higher rate in HIV-infected individuals. One of the reasons why HIV was not associated with a positive Xpert Ultra result could have been the low proportion of the HIV-infected participants (18.4%) in our study compared with the Gounden cohort (88%).

### Study limitations

The present study had a number of limitations. Firstly, the sample size was small. However, recruiting 165 patients for pleural biopsy is one of the largest studies evaluating same-day diagnostic tools for the diagnosis of pleural TB. It is challenging to carry out a large study but clearly this will need to be undertaken. Secondly, clinical variables studied were limited. However, this was a retrospective analysis and a future prospective study could collect variables such as presence of systemic lymphadenopathy, fever and history of positive TB contact. These pre-test variables might be important in determining selection of patients for Xpert Ultra testing.

## Conclusion

CXR features suggestive of active TB are significantly associated with a positive Xpert Ultra test result on pleural fluid. These data inform clinical practice in resource-poor settings.

## Figures and Tables

**TABLE 1:** Demographic, radiological and pleural fluid test characteristics (biochemical and microbiological tests) used in the univariate analysis

**Variables**	**Ultra-negative** (N=30), *n* (%)*	**Ultra-positive** **(N=19), *n* (%)**	***p*-value**
**Gender**			0.33
Male	18 (60)	14 (73.7)	
Female	12 (40)	5 (26.3)	
Age (mean), years	45.6	38.6	0.14
**Previous TB**			0.70
Yes	5 (16.7)	4 (21)	
No	25 (83.3)	15 (79)	
**HIV status**			0.25
Positive	4 (13.3)	5 (26.3)	
Negative	26 (86.7)	14 (73.7)	
CD4 count (mean), cells/µL	115.5	182.2	0.19
**CXR suggestive of TB**			0.01
Yes	3 (10)	8 (42)	
No	27 (90)	11 (58)	
**Pleural effusion size**			0.06
Small	12 (40)	9 (47.4)	
Moderate	15 (50)	4 (21.5)	
Large	3 (10)	6 (31.6)	
TTP (days)	23	18.8	0.09
Mean pleural fluid protein, g/L	54.9	63.5	0.01
Mean adenosine deaminase, U/L	48.5	88.5	0.001
**Pleural fluid TB culture**			0.71
Positive	22 (73.3)	13 (68.4)	
Negative	8 (26.7)	6 (31.6)	
Pleural fluid lymphocytes, ×10^9^	2.5	7.4	0.17

CXR = chest X-ray

TB = tuberculosis

TTP = time to positive culture

*Unless otherwise specified

**TABLE 2:** Multivariate analysis incorporating demographic, radiological and pleural fluid test characteristics (biochemical and microbiological tests): Model 1*

	**OR**	***p*-value**	**95% CI**
CXR suggestive of TB	12.33	0.22	0.22 - 681.31
Pleural effusion size	0. 34	0.16	0.07 - 1.54
TTP	1.00	0.98	0.88 - 1.13
Pleural fluid protein	1.12	0.19	0.94 - 1.33
Adenosine deaminase	1.06	0.08	0.99 - 1.13

OR = odds ratio

CI = confidence interval

CXR = chest X-ray

TTP = time to positive culture

*Including all characteristics with p<0.1

**TABLE 3:** Multivariate analysis incorporating only immediately ascertainable (demographic and radiological) characteristics: Model 2*

	**OR**	***p*-value**	**95% CI**
CXR suggestive of TB	4.48	0.021	1.25 - 16.04
Pleural effusion size	1.64	0.20	0.77 - 3.49

OR = odds ratio

CI = confidence interval

CXR = chest X-ray

*Including all characteristics with p<0.1

**TABLE 4:** Xpert Ultra result in participants with a CXR suggestive of active TB

**Xpert Ultra result**	**CXR suggestive of TB, *n* (%)**	**(95% CI)**
Negative	3 (27.3)	14.56 - 39.43
Positive	8 (72.7)	60.56 - 85.43

CXR = chest X-ray

CI = confidence interval
